# Patterns of homelessness and housing instability and the relationship with mental health disorders among young people transitioning from out-of-home care: Retrospective cohort study using linked administrative data

**DOI:** 10.1371/journal.pone.0274196

**Published:** 2022-09-02

**Authors:** Fadzai Chikwava, Melissa O’Donnell, Anna Ferrante, Eduwin Pakpahan, Reinie Cordier

**Affiliations:** 1 Curtin School of Allied Health, Curtin University, Perth, Western Australia, Australia; 2 Australian Centre for Child Protection, University of South Australia, Adelaide, South Australia, Australia; 3 Telethon Kids Institute, University of Western Australia, Nedlands, Western Australia, Australia; 4 Curtin School of Population Health, Curtin University, Perth, Western Australia, Australia; 5 Department of Mathematics, Physics & Electrical Engineering, Northumbria University, Newcastle upon Tyne, United Kingdom; 6 Department of Social Work, Education and Community Wellbeing, Northumbria University, Newcastle upon Tyne, United Kingdom; New York City Department of Health and Mental Hygiene, UNITED STATES

## Abstract

**Objectives:**

The study examined the relationship between mental health, homelessness and housing instability among young people aged 15–18 years old who transitioned from out-of-home in 2013 to 2014 in the state of Victoria, Australia with follow-up to 2018. We determined the various mental health disorders and other predictors that were associated with different levels of homelessness risk, including identifying the impact of dual diagnosis of mental health and substance use disorder on homelessness.

**Methodology:**

Using retrospective de-identified linked administrative data from various government departments we identified various dimensions of homelessness which were mapped from the European Topology of Homelessness (ETHOS) framework and associated mental health variables which were determined from the WHO ICD-10 codes. We used ordered logistic regression and Poisson regression analysis to estimate the impact of homelessness and housing instability respectively.

**Results:**

A total homelessness prevalence of 60% was determined in the care-leaving population. After adjustment, high risk of homelessness was associated with dual diagnosis of mental health and substance use disorder, intentional self-harm, anxiety, psychotic disorders, assault and maltreatment, history of involvement with the justice system, substance use prior to leaving care, residential and home-based OHC placement and a history of staying in public housing.

**Conclusions:**

There is clearly a need for policy makers and service providers to work together to find effective housing pathways and integrated health services for this heterogeneous group of vulnerable young people with complex health and social needs. Future research should determine longitudinally the bidirectional relationship between mental health disorders and homelessness.

## Introduction

### Homelessness rates

Homelessness is a significant global issue. In Australia, there is evidence of an increase in homelessness with the number of clients assisted by specialist homelessness services increasing from 279,200 in 2015/2016 to almost 290,500 in 2019/2020 [1]. The state of Victoria in Australia had the second highest rate of homelessness at 175 per 10,000 people, an increase from 42 per 10,000 people in 2016, with 57% of the homeless being under the age of 35 years [1].

Studies have shown that homelessness is one of the many negative outcomes experienced by young people leaving out-of-home care (OHC) [2, 3]. Homelessness rates among young people ageing out of care range from 20% to 40% [4–6]. In Australia, almost 8,800 children and young people from OHC received assistance during 2019/20 from the specialist homelessness services [1]. The risk of homelessness has been attributed to various factors such as poor transition planning from government departments and lack of suitable housing options [7].

### Specialist homelessness services in Australia

Since 2013, the Australian government made a commitment through the Specialist Homelessness Service System (SHSS) to provide almost 300,000 Australians each year with a range of support services to those at risk of or experiencing homelessness [8]. Through the National Housing and Homelessness Agreement (NHHA), which commenced in July 2018, state and territory governments committed to implementing a policy of ‘no exits into homelessness’ from institutions, which include those at risk of homelessness such as young people transitioning from OHC [9]. Data on the number of clients accessing Specialist Homelessness Services (SHS) in Australia provide some indication of the rate of homelessness or housing instability in Australia.

The number of clients assisted by specialist homelessness agencies increased at an average annual rate of 2.6% since 2011 [1], with the state of Victoria having the second highest number of people accessing SHS. The people accessing homelessness services were mostly female (60%), young people below the age of 18 years (33%), and those who identified as Aboriginal (27%). The main reasons stated for seeking SHS among young people from OHC were family and domestic violence (50%) and having a mental health problem (42%) [1]. This study utilises the Victorian portion of the national collection of homelessness services data from which these statistics are drawn.

### Types of homelessness

There is a lack of universally accepted definition of homelessness. Most definitions are premised on three domains relating to what constitutes a “home” and the lack of it is then an indicator of homelessness. This includes (i) absence of adequate housing; (ii) lack of privacy; and (iii) lack of legal entitlement to housing [10–12]. There has been increasing international attention on the need for a consistent definition of homelessness, hence in 2005 the European Federation of National Organizations Working with the Homeless (FEANTSA) launched a typology to define data collection on homelessness called ETHOS, the European Typology of Homelessness and Housing Exclusion [13–15], which is premised on the three domains defined above. This framework has been used in some countries to monitor homelessness and housing exclusion [13, 14], including the Australian Bureau of Statistics which draws heavily on the ETHOS definition.

Homelessness ranges from the most severe or highest risk form, commonly referred to as “rough sleeping” or primary homelessness [10, 12, 16], to temporary housing or secondary homelessness which involves people moving between various forms of temporary shelter such as emergency accommodation, staying with friends or supported accommodation [10, 12]. The least severe forms or tertiary homelessness may include non-conventional accommodation or sleeping in extremely overcrowded conditions [10, 12].

The ETHOS framework is comprised of four conceptual definitions (“rooflessness”, “houselessness”, “insecure housing” and “inadequate housing”) each of which was expanded to 13 operational definitions ranging from the most severe to the least severe form of homelessness. The last two ETHOS conceptual definitions do not refer to the literal form of homelessness (not having a roof over one’s head), but refer to individuals who may be at risk of homelessness.

The length of time that one spends being homeless or the number of homeless episodes also determines the severity of homelessness or instability in housing [17]. There is, however, some debate or disagreement on what constitutes short-term or long-term homelessness [18]. A number of studies have identified individuals as chronically homeless if they have experienced continuous homelessness for one year or more, or four or more episodes of homelessness in the last three years where the combined length of time being homeless on those occasions is at least 12 months [16]. Short‑term homeless has been defined as experiencing less than three months in duration of homelessness or medium-term homelessness, if they have experienced episodes of homelessness for 3 to 11 months [18]. Others use the term “episodically homelessness” to refer to frequent shifts between sheltered and unsheltered circumstances [19, 20].

### Risk and protective factors for homelessness

Risk factors for homelessness among young people leaving care comprise interrelated and dynamic individual, family, social and structural factors [21, 22]. These factors are both intrinsic and extrinsic (involving various groups) [23]. The background or family risk factors that put young people leaving OHC at risk of homelessness include family violence, family separations [24], poor relationships with caregivers or parents [25, 26], poverty [27], child maltreatment [2, 26], parental substance abuse [21], and previous homelessness [28]. Intrinsic risk factors include poor mental health and substance abuse problems [2, 25], poor in-care experiences [28, 29], leaving care early, running away from home or care [25], multiple OHC placements [6], living in group care settings or state shelter compared to living with a family member or relative [30] and criminal justice involvement [4].

Lack of in-care planning, and financial and social support may result in young people leaving care without sufficient knowledge or capacity to navigate the housing market [31]. These factors are worsened by structural forces such as lack of available low-cost housing, poor economic conditions, and insufficient mental health services [22, 32]. Protective factors, which reduce the odds of homelessness, include having a close connection with a family member or carer [25], staying in care until the age of 21 years [25], having completed high school education [5, 25], being employed, and access to social support [5]. Prevention of homelessness after leaving care requires adequate preparation and planning with support from family or carers, caseworkers, and housing service providers.

### Homelessness and mental health

Having a mental health disorder is one of the most significant risk factors for young people experiencing homelessness. Mental health prevalence of up to 75%, including alcohol and drug disorders, have been reported in previous studies among young people experiencing homelessness [19, 32]. In 2018–19, there were 603 Specialist Homelessness Service (SHS) clients per 100,000 population in the state of Victoria with a current mental health issue, which is much higher than the Australian national rate of 393 SHS clients per 100,000 population [9].

Research on the relationship between homelessness and mental health has been previously conducted [33, 34], however, this has not been investigated among young people transitioning from care. Young people who have entered OHC are at significant risk of mental health issues due to possible exposure to maltreatment and/or adverse social circumstances [7]. These young people who end up being homeless often have histories of mental health issues, which may reinforce and lengthen their episodes of homelessness [19, 31, 35]. Moreover, young people experiencing homelessness are at an increased risk of developing or experiencing worsened mental health issues [6, 36, 37] compared to young people with stable housing.

The evidence also shows that mental health disorders are unevenly distributed in people across the different types of homelessness. Young people with persistent periods of homelessness have higher rates of mental health disorders than young people who have recent or single episodes of homelessness [25]. There are variations in the type and severity of mental health disorders, ranging from alcohol and drug use, psychosis, depression, stress adjustment disorders, anxiety, and self-injury, including dual diagnosis of mental health and substance use disorders [38]. There is some evidence that suggests that dual diagnosis of substance use and mental health disorders is common among homeless young people [39, 40], however, this evidence has not been determined among young people transitioning from OHC.

The interaction between homelessness experienced by young people and mental health disorders further perpetuates the homelessness cycle, making it harder for young people to achieve housing stability. By examining the relationship between mental health disorders and homelessness in this leaving care group, we can determine whether certain types of mental health disorders are associated with risk of homelessness and housing instability.

### Gaps in the current evidence

While prior research among young people transitioning from care have documented a strong relationship between mental health and homelessness, some of these studies are qualitative in nature or used small sample sizes, thus making it difficult to draw inferences at a population level [34, 41]. Some studies have been conducted without a complete psychiatric assessment to profile participants’ mental health or have not explored the different mental health disorders associated with homelessness or housing instability. This limits the knowledge of specific mental health disorders associated with homelessness and, in some cases, may over-estimate the prevalence of certain psychiatric disorders [5, 42, 43]. Further, some studies involve young people who have a history of OHC involvement but have not investigated the relationship between mental health and homelessness among young people transitioning from care [44].

Homelessness is a multi-faceted construct that constitutes a much wider group than rough sleeping only. Previous studies have not examined the different types of homelessness or housing instability, which will invariably lead to an under-estimation of the prevalence of homelessness [4–6, 30, 43]. Young people transitioning from out-of-home care who experience housing instability are at increased risk of becoming homeless and, therefore, the definition of homelessness should encompass various domains of housing instability and episodes of homelessness.

The relationship between homelessness and mental health disorders are not well understood regarding young people transitioning from OHC. Linked administrative data offers opportunities for examining the different types of homelessness and mental health problems to fill these gaps. The lack of robust evidence in young people transitioning from care examining the relationship between homelessness and different types of mental health disorders suggests that additional research is required.

## Purpose of study

The current study examines the relationship between mental health and homelessness among young people who transitioned from care between 2013 and 2014 in the state of Victoria, Australia using linked administrative data with follow-up to 2018. The linked datasets enable the investigation of demographic, contextual and mental health risk factors for young people transitioning from care and their relationship to homelessness. Such evidence is vital to contribute to understanding the relationship between homelessness and mental health among this population group, but also to inform the design and focus of homelessness interventions.

The ETHOS framework used in our study has been used to classify homelessness in studies internationally [13, 45, 46] and therefore can be replicated in future research. By utilising this framework, our study provides a more nuanced understanding of homelessness by considering the spectrum of homelessness from rooflessness, at the severe end, to the less severe form of extreme overcrowding. The framework provides a more in-depth understanding of the construct and provides a more accurate estimation of the true prevalence of homelessness.

The overall goal of the study is to provide a holistic analysis of the relationship between mental health disorders and homelessness. Specifically, the following research questions will be addressed:

1. What are the different types and severity of homelessness experienced by young people transitioning from care (RQ1)?
2. What are the mental health disorders that are significantly associated with homelessness and housing instability among young people transitioning from care (RQ2)?
3. What other significant predictors are associated with homelessness and housing instability among young people transitioning from care (RQ3)?
4. Do young people with dual diagnosis of mental health and substance use have higher levels of homelessness compared to young people without a dual diagnosis (RQ4)?

## Methods

### Study population

The study comprised a retrospective cohort of 1,848 young people aged 15–18 years who left the Victorian OHC system in 2013–2014, with follow up until 2018.

### Data sources

The study used de-identified linked administrative data from various government departments and these included data from the health, justice, family violence, alcohol and drug use information systems and child protection data collections. De-identified datasets with a unique identification number for each individual were provided to the researchers from The Centre for Victorian Data Linkage (CVDL). Ethics for conducting the study was provided by the Curtin Human Research Ethics Committee (Ethics number HRE2021-0151) and as per normal practice with linked datasets, the need for consent was waived by the ethics committee because of the anonymised nature of the linked administrative data used. The datasets for the individuals were available from 2011 to 2018, which allowed the study to obtain historical data 2–3 years prior to leaving care and up to 4–5 years after leaving care. The analysis was conducted for the total follow-up period from the time young people were in care until the period after they left care.

### Study measures

The following measures were used in the analysis. A full description of all the variables and the data sources is shown S1 Table. The analysis comprised participants’ demographic characteristics, homelessness and mental health variables and control variables described below.

#### Participants’ characteristics

These were obtained from child protection data and they included participants’ age of leaving care, their gender, Indigenous status, and geographical location classified as either regional or urban area.

#### Outcome variable: Homelessness

The main data source for housing information and homelessness was the homelessness data collection, of which the specialist homelessness service collection (SHSC) data is part of. The SHSC collects information on an ongoing basis about people who may be referred to, or access the specialist homelessness services (SHS) agencies for housing assistance [1]. The homelessness data contained information on individuals who were either homeless or at risk of becoming homelessness. Due to limited SHSC data prior to 2015, other sources were used to obtain housing information. These included the Victorian admitted hospital patient data, emergency department, and alcohol and drug use data collections.

The definition of homelessness used in this study is based on the ETHOS four conceptual definitions of roofless, houseless, insecure and inadequate accommodation. It thus encompasses all young people who were homeless and those at risk of homelessness. The ETHOS framework [13] was used to map the various housing types to 13 operational definitions from the worst form of homelessness (rough sleeping) to the least severe form of homelessness (extreme overcrowding). These variables included housing situation at present, residential type, tenure type and reasons for seeking homelessness services. A detailed mapping of this data is shown in S2 Table. A comprehensive and continuous housing dataset was then constructed by merging data from the different data sources. Derived variables of homelessness were then constructed as shown in S1 Table.

**Fig 1:**
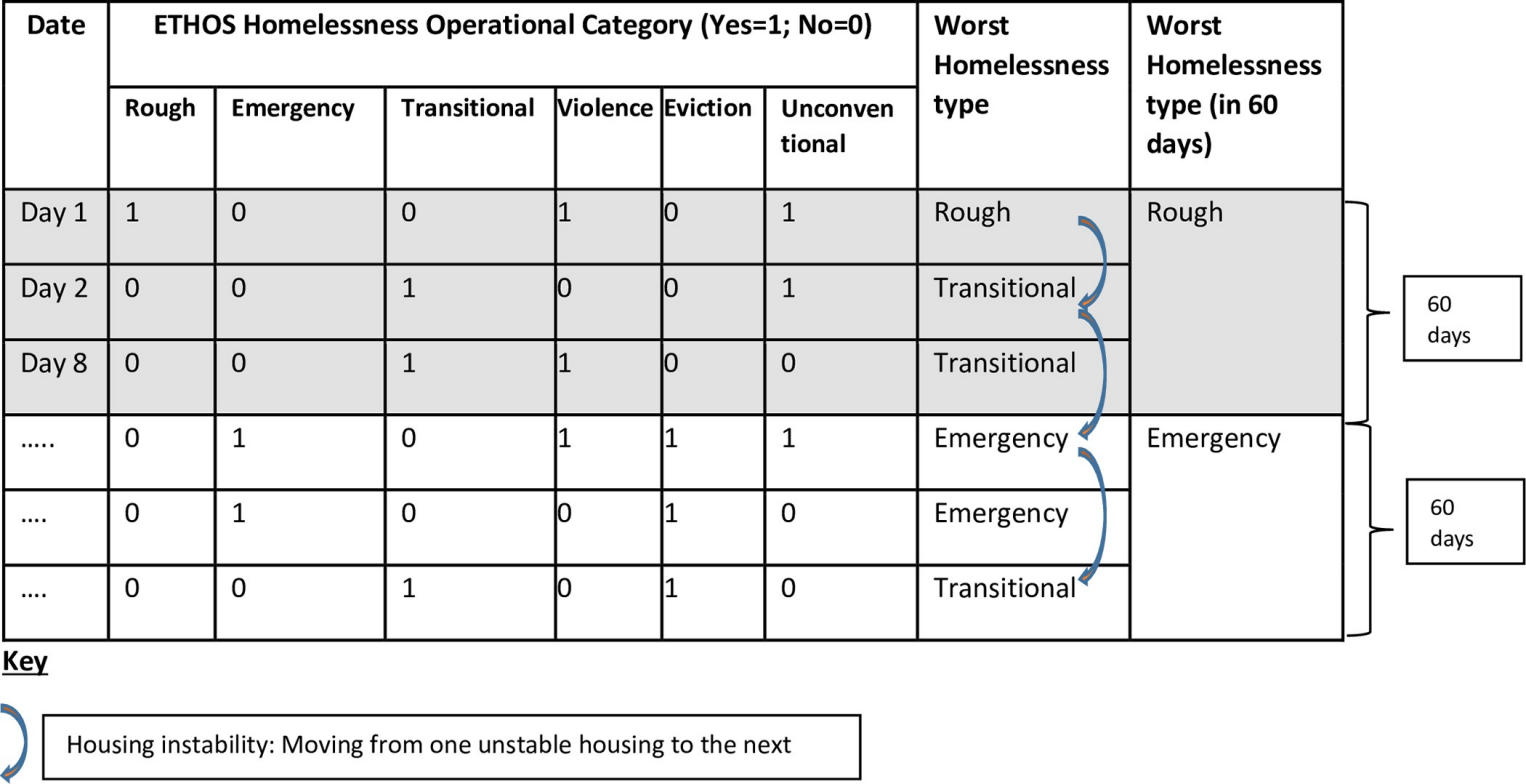
Construction of homelessness and housing instability variables.

Once the ETHOS homelessness type was determined for each episode from the combined dataset, the worst homelessness situation for that episode was then derived. This is consistent with the ABS report on defining homelessness [11] which recommends that to avoid double counting, where an individual could be classified in more than one homelessness category, it is advised to classify them in the category that is the highest on the hierarchy of homelessness (depicted in Fig 1 as the worst form of homelessness). Once the worst homelessness situation was determined, three key variables were constructed:

1. ***Homelessness outcome (binary)***
  If an individual had any record of homelessness based on the ETHOS framework the data was coded as one. The data was coded as zero if no evidence of homelessness was recorded from any of the housing data sources.
2. ***Homelessness risk score (continuous)***
  Records containing evidence of homelessness were considered to relate to the same ‘episode’ if they occurred within 60 days, otherwise they were considered to be separate episodes of homelessness. The 60 days was determined based on the average number of days that a new and different type of homelessness episode was recorded. A count of the different types of homelessness recorded was then conducted (Fig 1). Once the total for each of the operational categories of rough sleeping, emergency accommodation up to extreme overcrowding was recorded, we assigned a risk score for each category whereby the highest score was given to the worst form of housing. For instance, we assigned a score of 13 to rough sleeping, followed by emergency housing with a score of 12 and the least score was assigned to the least severe form of housing (i.e., overcrowding which had a score of 1). We then divided the total homelessness score by the total number of time periods (years) when homelessness was recorded. The final outcome variable was constructed as shown below (Table 1) to form a continuous homelessness risk score, whereby the higher the score the higher the risk profile of homelessness.
3. ***Housing Instability (Frequency Count)***
  Housing instability was defined as movement from one unstable housing situation or worst homelessness type to the next. This could mean having the first recorded episode as rough sleeping and the next recorded episode could be emergency accommodation, regardless of the date or duration from the first to the next episode. The curved arrows depict this movement from one worst homelessness type to the next, which could be any homelessness type as defined in the 13 ETHOS homelessness operational categories (Fig 1). A count of the number of movements was then obtained over the total follow-up period. The count ranged from 0 (= No movement) to a maximum of 46 movements (= very unstable). This variable was used in the Poisson regression analysis to determine the risk factors for housing instability.

**Table 1:** Calculation of homelessness risk score.

**Homelessness risk score =** (Tot_R * 13) + (Tot_E * 12) + (Tot_H * 11) + ……………………. . .+ (Tot_O *1) **N**
***Key*:** ***Tot_R** = Total episodes of Rough sleeping* ***Tot_E** = Total episodes of Emergency shelter housing* ***Tot_H** = Total episodes of accommodation for the homeless* ***………*** ***………*** ***Tot_O** = Total episodes of extreme overcrowding* ***N** = Total number of time periods (years) when homelessness was recorded*

#### Exposure variable: Mental health disorders

Information about mental health disorders were obtained from the following sources:

- The Victorian Admitted Episodes (hospital admissions) data which contains data on all public and private in-patient hospital admissions in Victoria, including detailed information on diagnosis and cause, along with information on separation type and referrals.
- The Victorian Emergency Department data contains information on all emergency department (ED) presentations at Victorian public hospitals and includes symptom, diagnosis and cause information where available for each presentation, along with departure and referral information.
- The Clinical Mental Health data contains information on clinical public mental health services provided in Victoria. The data includes both summaries of the services provided for each patient along with information on each individual contact with the mental health system (both inpatient and outpatient).

These sources contain diagnostic information based on the WHO International Classification of Diseases (ICD 10), recorded for each episode of care. S4 Table shows a list of these mental health disorders and corresponding ICD10 codes. Mental health disorders were combined from the above data sources and classified in four ways.

1. The first was a binary indicator of any mental health diagnosis or admission (including substance use disorder) (yes or no).
2. The second was by type of mental health disorder (diagnosis or admission) coded (yes or no) with eleven groups (outlined below) which were non-exclusive (young people with more than one diagnosis or admission could be counted in more than one mental health group):
  Substance use mental and behavioural disorders; (ii)Schizophrenia and psychoses (including manic/ bipolar disorders); (iii) Mood (affective) or depressive disorders; (iv) Severe stress and adjustment disorders; (v) Anxiety disorders; (vi) Personality disorders; (vii) Disorders of psychological development or behavioural and emotional disorders with onset usually occurring in childhood and adolescence; (viii) Intellectual disability; (ix) Self-harm (intentional); (x) Other substance use disorders (including poisoning by narcotics and psychodysleptics, anaesthetics, therapeutic gases and psychotic drugs and alcohol); (xi) Other mental health (behavioural syndromes associated with physiological disturbances and physical factors including organic mental disorder and eating disorders,)
3. The third variable was a derived dual diagnosis variable which consisted of the following four mental health categories: (i) No mental health admission or diagnosis recorded; (ii) Substance use only; (iii) Mental health only (no substance use); (iv) Mental health and substance use
4. The fourth was a categorical variable with four levels constructed for each of the nine mental health disorders as a mental health admission or diagnosis only, classified as follows: (i) No mental health admission or diagnosis recorded; (ii) Mental health diagnosis only (Clinical Mental Health or ED data); (iii) One mental health hospital admission; (iv) Two or more mental health hospital admissions.

Double counting from the Admitted episodes data and clinical mental health data was avoided by taking the highest number of admissions recorded from either data source.

#### Covariates

Based on previous literature the following variables were evaluated as potential predictors of homelessness and housing instability given, they have an influence on the outcome of interest in the analysis:

***Socio-demographic characteristics*:** These include gender, age when leaving OHC, indigenous status and region.

***Child Protection Involvement*:** The data included information on the last placement date, allegations, substantiations and information on care placements for all closed cases. Substantiated child maltreatment allegations included physical, sexual, psychological and child abandonment. Placement types included kinship care, residential care, general home-based care, complex or intensive home-based care and permanent care.

***Alcohol and Drug use involvement*:** We obtained data pertaining to assessment, treatment and support services provided to young people in our cohort who had alcohol and/or drug use problems prior to leaving care. Any involvement with these services was coded as one and no involvement was coded as zero.

***Assault and Maltreatment*:** The variables relating to abuse and maltreatment were obtained from the Child Protection and the Victorian admitted episodes data, Victorian emergency management data and clinical mental health datasets, where ICD10 codes relating to any form of assault were recorded. Any recorded instance of assault from these data sources was coded as one and zero if there was no recorded instance of assault.

***Family Violence*:** The family violence dataset contains information on services provided to both victims and perpetrators of family violence. The control variable was whether a young person was a perpetrator of violence prior to leaving care. Any recorded instance of violence from this data was coded as one, and zero if there was no recorded instance.

***Youth Justice*:** The youth justice dataset contains information on all criminal court orders in the youth justice system in Victoria. Variables extracted and utilised, were custodial or community justice involvement prior to leaving care. Any recorded instance of youth justice involvement from this data was coded as one, and zero if there was no recorded instance.

***Housing Integrated Information Program*:** This dataset contains information on Victorian public housing including applications for housing, tenancies, funding support for tenancies, and income sources used to pay rent. The main variable utilised was whether young people had a public housing tenancy as a dependent or main applicant prior to leaving care. Any recorded instance of public housing tenancy from this data was coded as one, and zero if there was no recorded instance.

### Statistical analysis

#### RQ1

Initially we conducted descriptive analysis of the prevalence of mental health disorders and homelessness. We examined the distribution of continuous variables and reported on mean scores where the distribution was normal and median scores where the distribution of continuous variables was skewed and there were large variances. Since the homelessness risk score outcome variable was highly skewed, a categorical variable was created which was used in the bivariate and multivariable analysis. The homelessness outcome variable was categorised into 4 groups with almost equal number of respondents in each group (not homeless, low, medium, and high).

#### RQ2 and RQ3

We then conducted ordered logistic regression analysis which is appropriate for ordered categorical data to predict homelessness risk. Bivariate regression analysis was conducted to determine the association between each predictor variable and the homelessness outcome variable. All covariates that were statistically significant or approached significance at *p<0*.*10* in the bivariate analysis were included in the multivariable analysis.

The ordered logistic regression analysis has to satisfy the proportional odds assumption or parallel regression assumption test [47, 48]. That is, the relationship of predictors to the odds of a response being in the next higher order category is the same regardless of which category is being compared. We therefore conducted the approximate likelihood-ratio test of proportionality of odds across response categories. A significant test statistic provides evidence that the parallel regression assumption has been violated (i.e., *p<0*.*05*) [47]. We adjusted for all socio-demographic and background characteristics as potential confounders in our models. Due to this large number of predictor variables (16 in total), a forward stepwise regression procedure was followed so that after each step in which a variable was added, all candidate variables in the model were checked to see if their significance had been reduced below a specified tolerance level of *p-value* of 0.1. This method allowed us to determine the variables which improved the fit of the model [49].

The second part of the research question was addressed using Poisson regression analysis to model the predictors of housing instability because the response variable is a count variable. Scaling of standard errors was conducted to account for over dispersion of the data. The last model on housing instability was restricted to young people who were homeless since housing instability was determined among this sub-population of homeless young people.

#### RQ4

Ordered logistic regression analysis was used to model the impact of dual diagnosis of any mental health disorder and substance use on the homelessness outcome.

For all models, the level of significance was set at a *p-value* of 0.05 and 95% confidence intervals are presented for all estimates. Maximum Likelihood Estimation (MLE) was used to estimate parameters in our model. Sensitivity analysis was conducted to determine homelessness prevalence from each data source to validate the other data sources as proxy data for homelessness. Missing data on predictor variables was handled using the list-wise deletion method. The data was analysed using STATA 14.2.

## Results

### Descriptive analysis

#### Homelessness patterns

Our total cohort of 1,848 young people comprised more females (55%) than males (45%) and more left care at a younger age of 15–16 years old (55%) compared with those who left care aged 17–18 years old (45%). The majority of the cohort was mostly non-indigenous (82%) compared to indigenous young people (18%). We determined a total homelessness prevalence of 60% from the merged datasets which includes those who are literally homeless and those at risk of homelessness as per ETHOS definition. This prevalence was significantly higher (*t = 425; P<0*.*001* as compared to the homelessness prevalence (40%) of young people (15–24 years old) in the state of Victoria [1]. The main data source for homelessness was the Victorian homelessness data collection, which identified that 57% of young people were or had been homeless in the study period. The other data sources identified the following homelessness numbers: Alcohol and Drug Information System (15%), Victorian Emergency Management Dataset (10%) and Victorian Admitted Episodes (3%). The young people had a median of four homelessness episodes (Table 2).

**Table 2:** Demographic and background characteristics for total cohort, homelessness and housing instability summaries and risk scores.

Independent Variables		Total Cohort		Homeless N (%)	Chi-Squared test statistic (*p-value* ^*1*^)	Total homelessness episodes: Mean (SD)	Homelessness risk score: Mean (SD)	Housing Instability Mean (SD)
		N	(%)					
**TOTAL**		1, 848	(100%)	1,111 (60.1%)		4.1 (3.3)	13.2 (7.6)	3.3 (4.8)
**Sex**	Male	841	(45.5%)	492 (58.5%)	1.7 (p = 0.194)	3.8 (3.1)	13.6 (7.8)	2.7 (4.4)
	Female	1,007	(54.5%)	619 (61.5%)		4.4 (3.4)	12.8 (7.5)	3.7 (5.1)
**Age when leaving OHC**	15–16 years	1,016	(55.0%)	652 (64.2%)	15.5 (p<0.001)	3.9 (3.0)	12.6 (6.9)	2.9 (4.1)
	17–18 years	832	(45.0%)	459 (55.2%)		4.5 (3.6)	13.9 (8.5)	3.7 (5.7)
**Indigenous Status**	Indigenous	333	(18.0%)	259 (77.8%)	52.8 (p<0.001)	4.8 (3.6)	14.0 (8.3)	4.1 (5.7)
	Non-indigenous	1,515	(82.0%)	852 (56.2%)		3.9 (3.2)	12.9 (7.4)	3.0 (4.5)
**Region**	Major cities	1,170	(63.3%)	683 (58.4%)	4.0 (p = 0.044)	4.3 (3.5)	13.6 (7.8)	3.5 (5.3)
	Regional areas	678	(36.7%)	428 (63.1%)		3.8 (2.9)	12.4 (7.2)	2.8 (3.9)
**Type of OHC**	Kinship care	664	(35.9%)	333 (50.1%)	100.8 (p<0.001)	3.4 (2.7)	11.5 (6.7)	2.5 (3.7)
	HBC-General	313	(16.9%)	215 (68.7%)		4.2 (3.2)	13.2 (6.7)	3.0 (4.0)
	HBC-Complex/Intensive	176	(9.5%)	103 (58.5%)		3.8 (3.1)	13.5 (9.4)	3.3 (5.6)
	Permanent care	47	(2.5%)	15 (31.9%)		2.5 (1.7)	11.3 (6.1)	1.1 (1.2)
	Residential care	560	(30.3%)	417 (74.5%)		4.9 (3.7)	14.5 (7.9)	4.1 (5.7)
	Other/not specified	88	(4.8%)	-		3.1 (3.2)	11.5 (9.2)	2.6 (4.3)
**Child Protection: History of Abuse and Maltreatment**	Psychological abuse	1, 057	(57.2%)	696 (65.8%)	33.8 (p<0.001)	4.3 (3.4)	13.5 (7.8)	3.4 (5.2)
	Physical abuse	615	(33.3%)	378 (61.5%)	0.7 (p = 0.405)	4.2 (3.1)	13.3 (7.8)	3.2 (4.4)
	Sexual abuse	162	(8.8%)	90 (55.6%)	1.5 (p = 0.214)	4.0 (3.1)	13.0 (6.8)	3.0 (4.4)
	Physical development	157	(8.5%)	102 (65.0%)	1.7 (p = 0.195)	3.9 (3.1)	13.4 (6.1)	2.9 (3.5)
**Assault and Maltreatment from Hospital Data**	Physical assault	234	(12.7%)	193 (82.5%)	55.9 (p<0.001)	5.2 (3.9)	15.1 (8.4)	4.6 (6.5)
	Sexual assault	223	(12.1%)	158 (70.9%)	12.2 (p<0.001)	5.0 (3.7)	14.8 (8.9)	4.7 (6.2)
	Family violence perpetrator	72	(3.9%)	51 (70.8%)	3.6 (p = 0.060)	3.9 (2.8)	13.4 (6.2)	2.6 (2.9)
**Alcohol & Drug use prior leaving OHC**		435	(23.5%)	354 (81.4%)	107.3 (p<0.001)	5.3 (3.8)	15.1 (7.8)	4.6 (6.0)
**Community justice involvement prior leaving OHC**		376	(20.3%)	304 (80.8%)	84.6 (p<0.001)	5.1 (3.9)	14.5 (7.3)	4.2 (5.8)
**Custodial justice involvement**		207	(11.2%)	174 (84.1%)	55.7 (p<0.001)	5.4 (3.8)	15.2 (7.2)	4.2 (5.9)
**Public Housing tenant prior leaving OHC**		482	(26.1%)	341 (70.8%)	30.7 (p<0.001)	4.2 (3.5)	12.8 (7.3)	3.3 (5.1)

*Note*: OHC = Out-of-home Care; HBC = Home-based care; SD = Standard Deviation

1 Chi-square test for significant difference among homeless vs. not homeless young people

A significantly higher prevalence of homelessness was reported among young people who left care aged 15–16 years old (64%), Indigenous young people (78%), people from regional areas (63%), young people who stayed in residential care (74%), and among those who experienced any form of abuse or maltreatment (>70%). Higher prevalence was also reported among those who had a history of involvement with the community justice (81%), custodial justice (84%), prior substance abuse problems (81%) and those who had stayed in public housing in the past (71%).

The homelessness risk score among those who were homeless ranged from 3 to 65, showing great variability in homelessness risk (Mean 13.2, SD 7.62). The highest homelessness risk scores (14 and above) and highest housing instability (4 or more homelessness type changes) were among young people who were: Indigenous, lived in residential care, experienced physical assault and sexual assault, involved in substance abuse prior to leaving care, and involved with community or custodial justice prior to leaving care.

**Fig 2:**
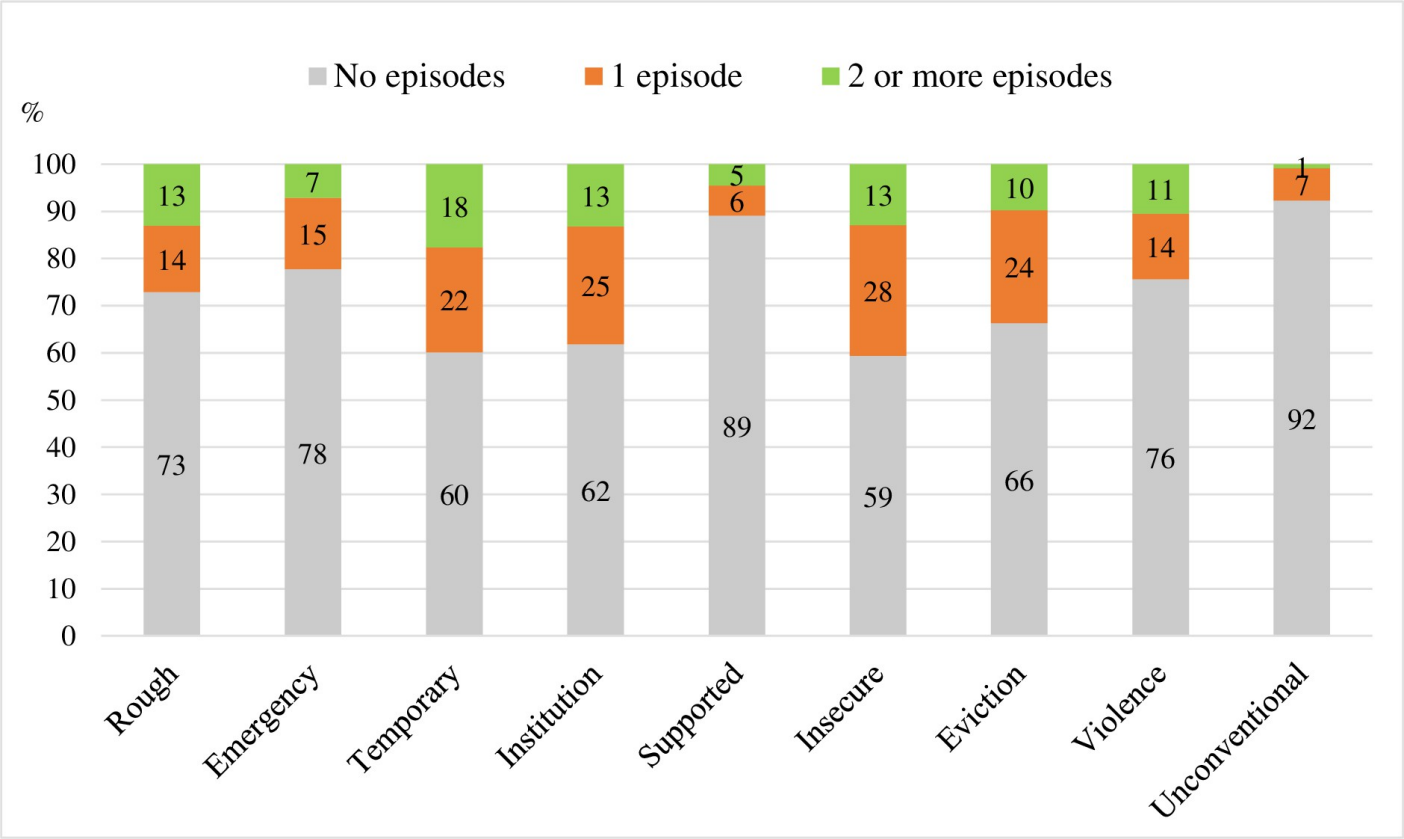
Proportion of homeless young people who experienced various forms of homelessness types (2013–2018).

The most common homelessness types experienced were insecure (41%), transitional supported housing (40%), leaving institutions (38%) or facing threat of eviction (34%) (Fig 2). The homelessness types where the highest multiple episodes were recorded were temporary or transitional supported housing (18%), leaving institutions (13%) and insecure accommodation (13%) (Fig 2).

#### Homelessness and mental health

There were varied mental health diagnostic groups recorded for young people and the young people could be grouped into one or more diagnostic groups. Overall, 61% of the sample had any form of mental health diagnosis or substance use disorder from the time young people were in OHC to the time they left care in the total follow up period. However only 623 (34%) young people had mental health disorders after they left OHC. The most common forms included substance use disorder (32%), childhood and psychological development disorders (31%), intentional self-harm (31%), mood disorders (25%), and depression (25%). Dual diagnosis of any mental health disorder and substance disorder was 27% (Table 3).

**Table 3:** Mental health characteristics for total cohort, homelessness and housing instability summaries and risk scores.

Mental Health Disorder	Total Cohort	Homeless N (%)	Chi-Squared test statistic (*p-value*^*1*^)	Total homelessness episodes Mean (SD^2^)	Homelessness risk score Mean (SD)	Housing Instability Mean (SD)
	N (%)					
No mental health disorder	716 (38.7%)	280 (39.1%)	215.2 (p<0.001)	3.0 (2.5)	11.3 (6.3)	1.9 (2.7)
Mental health or substance use	1,132 (61.3%)	831 (73.4%)	215.2 (p<0.001)	4.5 (3.4)	13.8 (7.9)	3.7 (5.3)
Mental health and substance use	500 (27.1%)	423 (84.6%)	171.3 (p<0.001)	5.3 (3.9)	15.6 (8.8)	4.9 (6.5)
Mental health due to substance use only	91 (4.9%)	66 (72.5%)	6.1 (p = 0.013)	3.8 (2.8)	12.8 (5.2)	2.9 (4.1)
Mental health only, no substance use	541 (29.3%)	342 (63.2%)	3.1 (0 = 0.080)	3.6 (2.7)	11.7 (6.5)	2.4 (3.1)
Psychosis	226 (12.2%)	189 (83.6%)	59.4 (p<0.001)	5.5 (4.1)	16.4 (9.1)	5.0 (7.1)
Childhood and psychological development	567 (30.7%)	435 (76.7%)	94.0 (p<0.001)	4.8 (3.7)	14.5 (8.5)	4.2 (6.0)
Substance use disorder	586 (31.7%)	485 (82.8%)	185.4 (p<0.001)	5.1 (3.8)	15.3 (8.4)	4.7 (6.2)
Mood or depression	465 (25.2%)	365 (78.5%)	87.5 (p<0.001)	4.9 (3.7)	14.6 (8.1)	4.2 (6.0)
Anxiety	321 (17.4%)	257 (80.1%)	64.4 (p<0.001)	5.1 (3.9)	15.1 (8.4)	4.7 (6.6)
Stress and adjustment	463 (25.1%)	357 (77.1%)	74.3 (p<0.001)	5.1 (3.8)	15.1 (8.6)	4.5 (6.2)
Personality	350 (18.9%)	293 (83.7%)	100.3 (p<0.001)	5.1 (3.8)	14.9 (8.3)	4.7 (6.5)
Intellectual disability	105 (5.7%)	77 (73.3%)	8.1 (p = 0.004)	5.0 (4.2)	14.2 (8.1)	4.2 (6.5)
Intentional self-harm	576 (31.2%)	462 (80.2%)	140 (p<0.001)	5.0 (3.8)	14.8 (8.4)	4.5 (6.0)
Substance use (other)	415 (22.5%)	336 (81.0%)	97 (p<0.001)	5.2 (3.9)	15.1 (8.1)	4.7 (6.4)
Mental Health (other^3^)	233 (12.6%)	193 (82.2%)	57 (p<0.001)	5.6 (4.1)	16.1 (9.1)	5.3 (7.3)

Note

1 Chi-square test for significant difference among homeless vs. not homeless young people

2 SD = Standard Deviation

3 Behavioural syndromes associated with physiological disturbances and physical factors

Very high prevalence of homelessness was experienced by young people with any form of mental health disorder ranging from 72% to 85%. The highest prevalence rate of homelessness was among young people who had dual diagnosis mental health and substance use disorder (85%). Young people with psychotic disorders had the highest number of homelessness episodes (Mean 5.5, SD 4.1). The worst forms of homelessness were experienced among those who had psychotic disorders (risk score 16.4), dual diagnosis mental health and substance use disorders (risk score 15.6), substance use disorder (risk score 15.3), anxiety (risk score 15.1), stress and adjustment disorders (risk score 15.1), intentional self-harm (risk score 14.8) and personality disorders (risk score 14.9). The same pattern of mental health disorders was experienced for high housing instability scores (Table 3).

### Multivariable analysis

#### Predictors of homelessness

The assumption of the approximate likelihood-ratio test of proportionality of odds across response categories for the ordered logistic regression analysis was satisfied (*X*^2^(32) 42, *p* = 0.11). In the bivariate analysis, each predictor was significantly associated with the odds of high homelessness risk scores, however, this association was attenuated after controlling for background and demographic characteristics. In the multivariable analysis, significant mental health predictors of high homelessness risk scores were substance use disorder (Adjusted Odds Ratio (aOR): 1.97, 95% CI: 1.57–2.47), intentional self-harm (aOR: 1.57, 95% CI: 1.23–2.02), anxiety (aOR: 1.48, 95% CI: 1.14–1.92), and borderline significance for psychotic disorders (aOR: 1.35, 95% CI: 0.99–1.83) (Table 4).

**Table 4:** Ordered logistic regression analysis for predictors of homelessness risk.

Characteristic	Homeless N (%)	Bivariate Analysis		Multivariable Analysis	
		Crude OR (95% CI)	*P-value*	Adjusted OR (95% CI)	*P-value*
**Mental Health Disorder**					
Psychosis	189 (83.6%)	3.29 (2.56 to 4.24)	<0.001	1.35 (0.99 to 1.83)	0.056
Stress and adjustment	357 (77.1%)	2.81 (2.31 to 3.41)	<0.001	1.15 (0.89 to 1.47)	0.286
Anxiety	257 (80.1%)	2.79 (2.25 to 3.48)	<0.001	1.48 (1.14 to 1.92)	0.003
Substance use disorder	485 (82.8%)	4.66 (3.86 to 5.61)	<0.001	1.97 (1.57 to 2.47)	<0.001
Mood or depression	365 (78.5%)	2.89 (2.38 to 3.50)	<0.001	1.27 (0.98 to 1.65)	0.072
Personality disorder	293 (83.7%)	3.32 (2.69 to 4.11)	<0.001	1.15 (0.86 to 1.53)	0.350
Intellectual disability	77 (73.3%)	1.84 (1.29 to 2.62)	0.001	0.91 (0.61 to 1.36)	0.643
Self-harm	462 (80.2%)	3.73 (3.10 to 4.48)	<0.001	1.57 (1.23 to 2.02)	<0.001
**Indigenous status**					
Non-indigenous	852 (56.2%)	*Reference*		*Reference*	
Indigenous	259 (77.8%)	2.46 (1.98 to 3.05)	<0.001	2.24 (1.77 to 2.83)	<0.001
**Age of leaving care**					
15–16 years	652 (64.2%)	*Reference*		*Reference*	
17–18 years	459 (55.2%)	0.81 (0.68 to 0.96)	0.013	0.85 (0.71 to 1.02)	0.086
**Abuse and Maltreatment**					
Physical assault victim	193 (82.5%)	3.06 (2.38 to 3.92)	<0.001	1.45 (1.10 to 1.92)	0.009
Psychological harm victim	696 (65.8%)	1.67 (1.41 to 1.98)	<0.001	1.26 (1.04 to 1.51)	0.016
Community justice involvement	304 (80.8%)	3.36 (2.72 to 4.14)	<0.001	1.58 (1.23 to 2.04)	<0.001
Substance use prior to leaving care	354 (81.4%)	3.88 (3.17 to 4.75)	<0.001	1.69 (1.33 to 2.16)	<0.001
**Last OHC placement type**					
Kinship care	333 (50.1%)	*Reference*		*Reference*	
HBC-General	215 (68.7%)	2.21 (1.73 to 2.83)	<0.001	2.15 (1.66 to 2.79)	<0.001
HBC-Complex/Intensive	103 (58.5%)	1.39 (1.03 to 1.88)	0.033	1.43 (1.04 to 1.97)	0.029
Permanent care	15 (31.9%)	0.46 (0.25 to 0.85)	0.013	0.72 (0.38 to 1.37)	0.321
Residential	417 (74.5%)	3.08 (2.50 to 3.80)	<0.001	1.75 (1.39 to 2.20)	<0.001
Public housing tenant prior OHC	341 (70.8%)	1.56 (1.29 to 1.87)	<0.001	1.47 (1.21 to 1.80)	<0.001

*Note*: OR = Odds Ratio; OHC = Out-of-home care; HBC = Home-based care; 95% CI = Confidence Interval

The odds of a high homelessness risk score versus the combined medium, low and not homeless categories were 1.97 times greater among young people with a substance use disorder compared with those not having a substance use disorder. Likewise, the odds of the combined high and medium risk categories versus the low and not homeless categories was 1.97 times greater among young people with a substance use disorder compared with those not having a substance use disorder. This interpretation applies to all other significant predictors of high homelessness risk scores.

Indigenous young people had double the odds of being in the higher homelessness categories versus being in the lower/not homeless categories (aOR: 2.24, 95% CI: 1.77–2.83). Other significant predictors were physical assault (aOR: 1.45, 95% CI: 1.10–1.92), psychological harm (aOR: 1.26, 95% CI: 1.04–1.51), history of involvement with community justice (aOR: 1.58, 95% CI: 1.23–2.04) and substance use prior to leaving care (aOR: 1.69, 95% CI: 1.33–2.16). A history of OHC residential placement was associated with higher odds of being in the high homelessness risk category compared with young people who stayed in kinship care (aOR: 1.75, 95% CI: 1.39–2.20). General home-based care was associated with higher odds of homelessness compared with kinship care (aOR: 2.15, 95% CI: 1.66–2.79) and complex home-based had higher odds of homelessness compared with kinship care (aOR: 1.43, 95% CI: 1.04–1.97). Young people with a history of staying in public housing had higher odds of homelessness compared to young people without any history of public housing tenancy (aOR: 1.47, 95% CI: 1.21–1.80).

In a separate model testing the significance of dual diagnosis of mental health and substance use disorders on homelessness risk, the odds of high homelessness risk scores were highest among young people with dual diagnosis (aOR: 5.31, 95% CI: 4.14–6.81) compared with those who only had one mental health disorder (aOR: 2.41, 95% CI: 1.93–3.01) or those with substance use only disorder (aOR: 2.70; 95% CI: 1.77–4.14) (Table 5).

**Table 5:** Ordered logistic regression analysis for dual diagnosis mental health and substance use predictors of homelessness risk.

Dual Diagnosis	Homeless N (%)	Bivariate Analysis		Multivariable Analysis	
		Crude OR (95% CI)	*P-value*	Adjusted^1^ OR (95% CI)	*P-value*
No mental health	716 (38.7%)	*Reference*		*Reference*	
Substance use only	91 (4.9%)	4.12 (2.77 to 6.12)	<0.001	2.70 (1.77 to 4.14)	<0.001
Mental health only	541 (29.3%)	2.67 (2.16 to 3.30)	<0.001	2.41 (1.93 to 3.01)	<0.001
Mental health & substance use	500 (27.1%)	8.30 (6.61 to 10.42)	<0.001	5.31 (4.14 to 6.81)	<0.001

*Note*: 1 Adjusted for placement type, age of leaving care, indigenous status, abuse and maltreatment, involvement with the community justice, history of alcohol and drug abuse and history of public housing tenant

OR = Odds ratio; CI = Confidence Interval

#### Predictors of housing instability

Young people with substance use disorder had a housing instability rate 1.44 times greater than those without any substance use disorder (IRR: 1.44, 95% CI: 1.24–1.67). Other significant predictors of housing instability were intentional self-harm (IRR: 1.23, 95% CI: 1.05–1.44), young people from Indigenous background (IRR: 1.24, 95% CI: 1.08–1.43), sexual assault victims (IRR: 1.25, 95% CI: 1.05–1.47), substance use prior leaving care (IRR: 1.30, 95% CI: 1.13–1.51), and those who stayed in residential OHC (IRR: 1.18, 95% CI: 1.00–1.39) (Table 6).

**Table 6:** Poisson regression analysis for predictors of housing instability.

Characteristic	Multiple moves N (%)	Bivariate Analysis		Multivariable Analysis	
		Crude IRR (95% CI)	*P-value*	Adjusted IRR (95% CI)	*P-value*
**Mental Health Disorder**					
Psychosis	120 (53.1%)	1.45 (1.24 to 1.70)	<0.001	1.04 (0.88 to 1.23)	0.616
Anxiety	163 (50.8%)	1.46 (1.27 to 1.69)	<0.001	1.11 (0.95 to 1.30)	0.175
Stress and adjustment	221 (47.7%)	1.52 (1.33 to 1.74)	<0.001	1.13 (0.97 to 1.32)	0.104
Substance use disorder	306 (52.2%)	1.83 (1.60 to 2.08)	<0.001	1.44 (1.24 to 1.67)	<0.001
Depression	219 (47.1%)	1.40 (1.22 to 1.60)	<0.001	0.95 (0.81 to 1.11)	0.483
Intellectual disability	42 (40.0%)	1.14 (0.89 to 1.47)	0.288	-	-
Self-harm	284 (49.3%)	1.64 (1.44 to 1.87)	<0.001	1.23 (1.05 to 1.44)	0.011
**Indigenous status**					
Non-Indigenous	420 (27.7%)	*Reference*		*Reference*	
Indigenous	156 (46.9%)	1.28 (1.10 to 1.49)	0.001	1.24 (1.08 to 1.43)	0.003
**Age of leaving care**					
15–16 years	330 (32.5%)	*Reference*		*Reference*	
17–18 years	246 (29.6%)	1.17 (1.02 to 1.33)	0.026	1.09 (0.96 to 1.25)	0.178
**Abuse and Maltreatment**					
Psychological harm victim	369 (34.9%)	1.09 (0.95 to 1.26)	0.216	-	-
Physical assault victim	125 (53.4%)	1.41 (1.21 to 1.66)	<0.001	1.14 (0.98 to 1.33)	0.099
Sexual assault victim	102 (45.7%)	1.39 (1.17 to 1.65)	<0.001	1.25 (1.05 to 1.47)	0.012
Custodial justice involvement	109 (52.7%)	1.27 (1.07 to 1.51)	0.006	0.94 (0.79 to 1.12)	0.549
Substance use prior to leaving care	221 (50.8%)	1.59 (1.39 to 1.82)	<0.001	1.30 (1.13 to 1.51)	<0.001
**Last OHC Placement Type**					
Kinship care	156 (23.5%)	*Reference*		*Reference*	
HBC-General	112 (35.8%)	1.20 (0.98 to 1.47)	0.077	1.17 (0.97 to 1.42)	0.108
HBC-Complex/Intensive	47 (26.7%)	1.14 (0.88 to 1.48)	0.322	1.08 (0.85 to 1.39)	0.523
Permanent care	6 (12.8%)	0.49 (0.20 to 1.21)	0.122	0.58 (0.25 to 1.37)	0.213
Residential	247 (44.1%)	1.47 (1.25 to 1.73)	<0.001	1.18 (1.00 to 1.39)	0.048
Public housing tenant prior leaving OHC	173 (35.9%)	1.00 (0.87 to 1.16)	0.936	-	-

*Note*: IRR = Incidence Rate Ratio; OHC = Out-of-home care; HBC = Home-based care; CI = Confidence Interval

## Discussion

### Homelessness prevalence and predictors of homelessness

This study is significant because it focuses on a particularly vulnerable group of young people going through significant developmental (social, physical, emotional, and cognitive) changes whilst transitioning to adulthood as well as transitioning from OHC. This is the first study globally, to determine the prevalence and diversity of homelessness types experienced by young people leaving OHC utilising retrospective linked data and applying the ETHOS framework [15]. The homelessness definition which extends beyond the literal definition of rough sleeping or rooflessness to the different forms, which include housing where there is no security or tenure and where the risk of ending up on the streets is high [14]. The multiple linked datasets provided background and personal characteristics associated with homelessness [50, 51] and the large sample size allowed for more accurate prediction of estimates.

The study shows a significantly higher prevalence of homelessness and housing instability (60%) among young people leaving OHC compared to young people (15–24 years old) in Victoria (40%) during the same time period [1]. This could be an under-estimation of the true prevalence of homelessness. Our study provides a more sensitive measure of homelessness. The authors acknowledge that homelessness among young people is likely to be underestimated in the Census statistics [52], for instance, young people who are homeless and ’couch surfing’, would have a usual residence reported on census night. Higher prevalence of homelessness was reported among those young people: aged 15–16 years when they left care (64%), who are of Indigenous descent (78%), from regional areas (63%), and whose last placement was residential care (74%). The results further suggest a diversity of homelessness experienced by young people ranging from rough sleeping, which had a prevalence of 27%, to less severe forms. The most common forms include living in temporary or transitional supported housing (40%), institutional housing (38%), insecure housing (41%), and housing where there is threat of eviction (34%).

### Homelessness and mental health

The prevalence of homelessness among those young people with a mental health disorder was over 20% higher (63%) compared with those without a mental health disorder (39%). The highest risk of homelessness was found to be among those who had a dual diagnosis of mental health and substance use disorders (85%), self-harm (80%), anxiety (80%), substance use disorders (83%), and psychosis (84%).

Our research provides compelling evidence of a strong relationship between mental health and homelessness. In addition to experiencing homelessness, care-leavers may be leaving care with diagnosed or undiagnosed mental health issues, including trauma. Substance use disorder, history of substance use, and dual diagnosis of substance use and mental health disorders emerged as significant predictors of homelessness. Young people with a dual diagnosis of mental health and substance use disorder often have complex and multifaceted needs. Some of the factors associated with dual diagnosis include longer homelessness duration, suicide attempts, engaging in risky sexual behaviour, and experiencing sexual and physical victimisation [53]. Furthermore, mental health and substance use disorders often interact to exacerbate each other. Alcohol and drugs are potentially taken as coping strategies among homeless young people who face high levels of adversity. The mental health of young people in care and transitioning from care requires sustained efforts to address complex health needs, support for young people to access health services and ongoing support for management and treatment of mental health disorders.

### Other predictors of homelessness

Other significant predictors included being Indigenous, having a history of residential care or home-based care placement, prior criminal justice involvement, those who experienced psychological or physical assault, having a history of substance abuse, and being a tenant in public housing prior to leaving care. Most of these results are consistent with previous research on risk factors for homelessness [2, 25, 31].

Indigenous young people experienced disproportionately high levels of homelessness with a 2.24 times greater risk of homelessness than non-Indigenous care leavers. Given the over-representation of Indigenous children in care, this should be seen as a priority issue for Indigenous young people transitioning from care. The results accord with those in the AIHW Homelessness report which recognises that Indigenous Australians continue to be over-represented in the homelessness population, as well as special homelessness services clients [1]. Indigenous Australians are already recognised as a national priority group in the National Housing and Homelessness Agreement (2018) and given that 78% of Indigenous care leavers in this cohort experienced homelessness, this should be recognised as a critical priority group for child protection and leaving care services.

## Implications for practice, policy, and research

### Policy or service delivery directions

While the study findings confirm the role of individual risk factors associated with pathways into homelessness, there is a need for policies and interventions to address structural factors that worsen individual risks and create barriers to exiting homelessness. To reduce the transiency and short-term housing problems, systems should be put in place to improve access and links to more sustainable and affordable longer term housing support where good quality standards can be maintained [54]. This requires effective coordination and collaboration among service providers. Young people often face long waiting lists to be approved for public housing [37]. The government needs to identify multiple approaches to increase funding and housing stock by developing a long-term, sustainable framework.

Service providers should try and minimise placement instability so that young people can build and maintain relationships with their carers while they are still in care. Our findings indicate that young people in kinship care arrangements had lower homelessness risk compared with those in residential care placement or home-based care placements, thereby supporting the notion that extended family support can reduce homelessness risk. For young people without kinship support, involving the young person in comprehensive transition planning is essential to imbue firm education and housing options as they transition from care [29].

Our study demonstrated a significantly higher prevalence of homelessness and housing instability among young people who left care at 15–16 years old compared to young people who left care at 17–18 years of age. A number of countries have either implemented or made commitments to implement policies and programs to extend the care of young people beyond 18 years of age [55, 56]. Extending the age of leaving care from 18 to 21 years old prevents young people from facing housing instability or becoming homeless and also leads to improvements in education, employment and training outcomes [56, 57].

Our findings highlight the complex needs of young people transitioning from care who have mental health issues and are at risk of homelessness. Service providers need to work collaboratively to provide holistic, co-ordinated and trauma informed care to address homelessness and mental health among young people transitioning from care. Integration of services is important, particularly in providing support for young people with a dual diagnosis of mental health and substance use. While this has been provided in most states in Australia, it is reported that service provision is ad-hoc and therefore there needs to be a more consistent and sustained approach in the service provision [7].

Due to heterogeneity among OHC leavers, a variety of programs which cater for specific needs of young people should be designed that integrate mental health and homelessness, including drug rehabilitation programs. Programs that can be adopted include the Housing First initiative, which have been successful in reducing the burden of homelessness, particularly among those with serious mental health problems [58, 59]. Early intervention is necessary to prevent the need for further services and the exacerbation of mental health and substance use challenges. Some programs have been shown to be effective in reducing hospital admissions, length of hospital stay and generating government cost savings [7, 60]. Central to all this, service providers should work collaboratively with the young person and their carers when planning interventions [61].

### Future research

The relationship between mental health and homelessness has been studied extensively, however, there is still a lack of evidence regarding the bidirectional relationship between mental health and homelessness and its potential mediating or moderating factors, particularly among young people transitioning from care. While young people may have mental health issues before becoming homeless, some mental health conditions may develop or worsen once they become homeless. Due to heterogeneity among this population group, further research is required to identify groups of young people in care who have pre-existing mental health conditions prior to their transition from care and compare their homelessness and mental health pathways to those young people who have no identified mental health conditions. This would provide greater evidence and understanding of the bi-directional relation between homelessness and mental health conditions in this high-risk group of young people [62]. In addition, a person-centred approach to the analysis may provide a better understanding of various sub-groups of young people transitioning from care, including those who are resilient.

Future research should investigate protective factors related to homelessness, such as having good relationships with families/ carers and social support. Moreover, it is also important to improve the evidence base for interventions that address mental health and homelessness issues that would result in improved outcomes for both Indigenous and non-Indigenous young people transitioning from care. Further research should ensure they use multiple data sources to augment or validate administrative data [50]. For instance, by conducting face-to-face interviews where young people could use a life event calendar to document places they lived, including the duration or time spent in those housing conditions and their quality of life [42].

## Limitations

This study has some limitations that could be addressed by future research. The homelessness data collection used in this study is a valuable source of longitudinal information, but only captures young people who may have been referred for services or those who attended services where homelessness was considered to have contributed to their mental health disorders. Therefore, we may have under-estimated the prevalence of homelessness, particularly for less visible homelessness such as couch surfing and insecure housing.

The mental health issues identified were those in which there was a diagnosis of a mental health condition or substance use disorder. This may have under-estimated mental health prevalence as young people in or transitioning from care may not have received a diagnosis or may be demonstrating symptoms of trauma which does not receive a diagnosis. By including variables such as abuse types, assault and exposure to family and domestic violence we hoped to capture the complexity of adversity which these young people may have been exposed to. While self-harm emerged as a significant predictor of homelessness, the findings should be interpreted with caution, given that some cases may not be accurately categorised [63].

While the study identified risk factors related to homelessness among young people transitioning from care, there are key protective factors which could not be analysed from this study due to absence of variables in the linked datasets such as social support, connection to an adult, family re-unification after OHC, education and employment status [25]. Other risk factors associated with homelessness which are not in our linked datasets include running away from OHC placements [25, 28] and placement instability [4, 30]. These additional factors are important to include in future research.

## Conclusion

The homelessness data collection and the multiple linked datasets provided housing, health, personal and background characteristics for the 1,848 young people who transitioned from care in the state of Victoria, Australia. The data allowed us to map the housing information against the 13 ETHOS homelessness categories and allowed us to report on the different forms of housing instability experienced by our population cohort, and so map their homelessness risk profiles. We determined the various mental health disorders that were associated with various levels of homelessness risk, including identifying dual diagnosis of mental health and substance use as the most significant predictor of homelessness.

Findings were consistent with previous studies; however, our study was able to determine the prevalence and diversity of homelessness among young people transitioning from care and the risk of homelessness associated with mental health disorders. There is clearly a need for policy makers and service providers in multiple sectors to work together to find lasting solutions for housing instability including offering integrated health services for this heterogeneous group of young people with multiple and complex health and social needs. This is a high priority given the impact of COVID-19 on young people obtaining affordable housing which has become scarce in recent years. Future research should determine longitudinally the bidirectional relationship between mental health disorders and homelessness and its potential mediators and moderators.

## Supporting information

S1 Table List of potential explanatory variables and outcomes for the study. (DOCX)

S2 Table Mapping of housing types variables to ETHOS framework. (DOCX)

S3 Table Prevalence of mental health disorders (Diagnosis and hospital admissions data). (DOCX)

S4 Table Mental health disorders and ICD-10 codes. (DOCX)
