# Supplementary material for: Patterns of homelessness and housing instability and the relationship with mental health disorders among young people transitioning from out-of-home care: Retrospective cohort study using linked administrative data
Source: PLoS One. 2022 Sep 2;17(9):e0274196. doi: 10.1371/journal.pone.0274196 (PMC9439254; doi:10.1371/journal.pone.0274196)
Supplement: S1 Table — (DOCX) [file pone.0274196.s001.docx]

**S1 Table. List of potential explanatory variables and outcomes for the study**

| **Variable** | **Description** | **Data Source** |
| --- | --- | --- |
| **Demographic** | | |
| atsi | As recorded at birth. 1=Aboriginal, 0=non-Aboriginal | Child Protection |
| sex | As recorded at birth. 1=male, 2=female | Child Protection |
| region | 1=Major cities, 2=regional/remote | Child Protection |
| ageatindex_cat | Age when one left OHC: 15=15-16 years, 17=17 – 18 years | Child Protection |
| **Maltreatment/abuse** | | |
| sexualabuse | 1=Sexual abuse, 0=not recorded | Child Protection |
| psychharm | 1=Psychological abuse, 0=not recorded | Child Protection |
| physdevharm | 1=Physical development harm, 0=not recorded | Child Protection |
| childabandoned | 1=Child abandoned, 0=not recorded | Child Protection |
| fmh_assault | Assault/ Maltreatment: As recorded using ICD-10 codes X85* X86* X87* X88* X89* X90* X91* X92* X93* X94* X95* X96* X97* X98* X99* Y0* T74* R456 Z61* Z62* | Victorian admitted episodes, Victorian emergency management and Clinical mental health datasets |
| **Recent OHC Placement type** | | |
| recent_place_type2 | Recent placement type: 0=Kinship care, 3=Home-based care general, 4=Home-based care complex/intensive, 7=Permanent care, 8=residential care, 99=not available/ not stated | Child Protection |
| **Violence** | |  |
| pre_fvperpetrator | 1=Perpetrator of family violence prior leaving care, 0=not recorded | Family violence |
| **Alcohol and drug use** | |  |
| preadis_user | 1=Had alcohol and drug treatment services prior leaving care, 0= not recorded | Alcohol and Drug information system |
| **Youth Justice involvement** | | |
| yjcustodialbeforeindex | 1=Had custodial justice recorded before leaving care, 0= not recorded | Youth Justice |
| yjcommunitybeforeindex | 1=Had community justice recorded before leaving care, 0= not recorded | Youth Justice |
| housingappprimarybeforeindex | 1=Had public housing application as a dependant or tenant prior leaving care, 0=not recorded | Housing Integrated Information Program |
| **Mental Health Disorders** | | |
| fmh_com_psych | Schizophrenia and psychoses | Victorian admitted episodes, Victorian emergency management and Clinical mental health datasets |
| fmh_com_dev | Psychological development or behavioural and emotional disorders with onset usually occurring in childhood and adolescence |  |
| fmh_stress | Severe stress-related disorders (including ptsd) |  |
| fmh_anxiety | Anxiety related disorders |  |
| fmh_depress | Mood or Depressive Disorders |  |
| fmh_person | Personality Disorders |  |
| fmh_disab | Intellectual Disability |  |
| fmh_selfharm | Self-harm |  |
| fmh_substdep | Substance use Disorder |  |
| fmh_substotherEX | Other Substance Use not stated above |  |

**S1 Table *cont’*. List of potential explanatory variables and outcomes for the study**

| **Variable** | **Description** | **Data Source** |
| --- | --- | --- |
| **Mental Health Disorders** | | |
| fmh_depress | Mood or Depressive Disorders | Victorian admitted episodes, Victorian emergency management and Clinical mental health datasets |
| fmh_person | Personality Disorders |  |
| fmh_disab | Intellectual Disability |  |
| fmh_selfharm | Self-harm |  |
| fmh_substdep | Substance use Disorder |  |
| fmh_substotherEX | Other Substance Use not stated above |  |
| fmh_depress | Other Mental Health including eating disorder and organic disorder |  |
| fmh_any | Any Mental Health (1=any mental health disorder incl. substance abuse, 0=no mental health recorded) |  |
| fmh_cormobid | Mental Health: 2=any mental health, 1=substance abuse only, 0=No mental health recorded |  |
| **Homelessness Outcome** | | |
| homeless_all (binary) | 1=homeless, 0=Homelessness not recorded | Homelessness Data Collection, Victorian admitted episodes, Victorian emergency management and alcohol and drug use information systems |
| tot_1rough_cat, tot_2emerg_cat, tot_3temp_cat, tot_6institution_cat, tot_7support_cat, tot_8insecure_cat, tot_9eviction_cat, tot_10violence_cat | **Total Episodes of each homelessness type:** Categorical variable: 0=No episodes recorded, 1=Only 1 episode recorded, 2+=2 or more episodes recorded | Homelessness Data Collection, Victorian admitted episodes, Victorian emergency management and alcohol and drug use information systems |
| homeless_outcome (continuous) | Product of total episodes and risk scores | Derived from above |
| homeless_outcome_CAT3 (categorical) | Categorical variable:  3=score of 37-222 (High)  2=score of 16-36 (Medium)  1=score of 3-15 (Low)  0=not homeless | Derived from above |
| **Housing Instability** | Movement from one unstable homelessness type to another:  Count variable (From 0=no movement to 46) |  |
