# Supplementary material for: Patterns of homelessness and housing instability and the relationship with mental health disorders among young people transitioning from out-of-home care: Retrospective cohort study using linked administrative data
Source: PLoS One. 2022 Sep 2;17(9):e0274196. doi: 10.1371/journal.pone.0274196 (PMC9439254; doi:10.1371/journal.pone.0274196)
Supplement: S2 Table — (DOCX) [file pone.0274196.s002.docx]

**S2 Table. Mapping of Housing Types variables to ETHOS Framework**

| **ETHOS** | | | | | **DATA SOURCE** | | |
| --- | --- | --- | --- | --- | --- | --- | --- |
|  | **Operational Category** | | **Living Situation** | | **Homelessness Data Collection** | **ADIS Data**  **(Variable: accommodation status)** | **Emergency Dept. Data (Variable: Type of usual accommodation)** |
| **ROOFLESS** | **1** | **People Living Rough** | **1.1** | **Public space or external space** | 1. Housing situation: Homeless - No shelter or improvised/inadequate dwelling 2. Residential type: No dwelling/street/park/in the open 3. Residential type: Motor vehicle | 1. Public place/temporary shelter/homeless | 1. Public Place (Homeless) |
|  | **2** | **People**  **in emergency accommodation** | **2.1** | **Night Shelter** | 1. Residential type: Emergency accommodation 2. Tenure type: Emergency accommodation/night shelter/women's refuge/youth shelter | N/A | Shelter/Refuge Other Than Homeless Shelter |
| **HOUSELESS** | **3** | **People in accommodation for the homeless** | **3.1** | **Homeless Hostel** | 1. Housing situation: Homeless - Short term temporary accommodation 2. Tenure type: Transition towards independent living/ boarding/rooming house 3. Residential Type: Boarding/ rooming house/hotel/motel/boarding school/ residential college | 1. Short-term crisis, emergency or transitional accommodation facility 2. Boarding house/private hotel | 1. Homeless persons shelter 2. Boarding/rooming/hostel |
|  |  |  | **3.2** | **Temporary Accommodation** |  |  |  |
|  |  |  | **3.3** | **Transitional supported accommodation** |  |  |  |
|  | **4** | **People in women’s shelter** | **4.1** | **Women’s shelter accommodation** | N/A | N/A | N/A |
|  | **5** | **People in accommodation for immigrants** | **5.1** | **Temporary accommodation/ reception centres** | N/A | N/A | N/A |
|  |  |  | **5.2** | **Migrant workers accommodation** | N/A | N/A | N/A |
|  | **6** | **People due to be released from institutions** | **6.1** | **Penal institutions** | 1. **Housing situation:** At risk of homelessness - Institutional settings 2. **Residential type:** Hospital/ Psychiatric unit/ Rehabilitation/ Adult correctional facility/ Youth/juvenile justice correctional centre 3. **Reason for seeking assistance:** Transition from: custodial arrangements/ foster care and child safety residential placements/ other care arrangements | a. Institutional setting (includes Residential aged care & Psychiatric/mental health community care)   1. Alcohol and Drugs Treatment Residence 2. Prison/remand centre/youth training centre | 1. Psychiatric hospital/ Other hospital setting/ prison/remand/ youth training centre |
|  |  |  | **6.2** | **Medical institutions** |  |  |  |
|  |  |  | **6.3** | **Children’s institutions/ homes** |  |  |  |
|  |  |  | **11.2** | **Non-conventional building** |  |  |  |
|  |  |  | **11.3** | **Temporary Structure** |  |  |  |

**S2 Table *cont’*. Mapping of Housing Types variables to ETHOS Framework**

| **ETHOS** | | | | | **DATA SOURCE** | | |
| --- | --- | --- | --- | --- | --- | --- | --- |
|  | **7** | **People receiving longer-term support (due to homelessness)** | **7.1** | **Residential Care for older homeless people** | 1. **Residential type** – Aged care facility/ Disability support | 1. Independent unit within retirement village 2. Supported accommodation or supported living facility NOT including Alcohol and Drug Treatment Residence | Residential Aged Care Facility/ |
|  |  |  | **7.2** | **Supported accommodation for formerly homeless people** |  |  |  |
| **INSECURE** | **8** | **People living in insecure accommodation** | **8.1** | **Temporarily with family/ friends** | 1. **Housing situation:** Homeless - House, townhouse or flat - couch surfer or with no tenure 2. **Reason for seeking assistance**: Itinerant | N/A | N/A |
|  |  |  | **8.2** | **No legal (sub) tenancy** |  |  |  |
|  |  |  | **8.3** | **Illegal occupation of land** |  |  |  |
|  | **9** | **People living under threat of eviction** | **9.1** | **Legal orders enforced (rented)** | 1. **Reason for seeking assistance:** financial difficulties/housing affordability stress/ housing crisis (eviction)/ previous accommodation ended/ unemployment/ problem gambling | N/A | N/A |
|  |  |  | **9.2** | **Re-possession orders (owned)** |  |  |  |
|  | **10** | **People living under threat of violence** | **10.1** | **Police recorded incidents** | 1. **Reason for seeking assistance:** sexual abuse/ domestic and family violence/ non-family violence | N/A | N/A |
| **INADEQUATE** | **11** | **People living in temporary/ non-conventional structures** | **11.1** | **Mobile Homes** | 1. **Residential type**: tent/ caravan/ cabin/ boat/ improvised building 2. **Tenure type**: caravan park 3. **Reason for seeking assistance:** Inadequate or inappropriate dwelling conditions | N/A | N/A |
|  |  |  | **11.2** | **Non-conventional building** |  |  |  |
|  |  |  | **11.3** | **Temporary Structure** |  |  |  |
|  | **12** | **People living in unfit housing** | **12.1** | **Occupied dwellings unfit for habitation** | N/A | N/A | N/A |

*^Note:^* ^N/A = No variables from the data source to map to ETHOS framework^
