# Supplementary material for: Patterns of homelessness and housing instability and the relationship with mental health disorders among young people transitioning from out-of-home care: Retrospective cohort study using linked administrative data
Source: PLoS One. 2022 Sep 2;17(9):e0274196. doi: 10.1371/journal.pone.0274196 (PMC9439254; doi:10.1371/journal.pone.0274196)
Supplement: S3 Table — (DOCX) [file pone.0274196.s003.docx]

**S3 Table. Prevalence of Mental Health Disorders (Diagnosis and Hospital Admissions Data)**

| **Mental Health Disorder** | **Total Cohort** | | **No Mental Health Admission/ Diagnosis**  **N (%)** | **CMH or ED Diagnosis only**  **N (%)** | **One Mental Health Hospital Admission**  **N (%)** | **Two or more Mental Health Hospital Admissions**  **N (%)** | **Total Hospital Length of Stay Median (SD)** |
| --- | --- | --- | --- | --- | --- | --- | --- |
|  | **N** | **(%)** |  |  |  |  |  |
| Mental health or substance use | 1,132 | (61.3%) | 716 (38.7%) | 477 (25.8%) | 265 (14.3%) | 390 (21.1%) | 5 (58.8) |
| Psychosis | 226 | (12.2%) | 1,622 (87.8%) | 102 (5.5%) | 49 (2.7%) | 75 (4.1%) | 14 (67.8) |
| Psychological development | 567 | (30.7%) | 1,281 (69.3%) | 391 (21.2%) | 83 (4.5%) | 93 (5.0%) | 5 (24.7) |
| Substance use disorder | 586 | (31.7%) | 1,262 (68.3%) | 244 (13.2%) | 211 (11.4%) | 131 (7.1%) | 2 (20.5) |
| Mood or depression | 465 | (25.2%) | 1,383 (74.8%) | 236 (12.8%) | 120 (6.5%) | 109 (5.9%) | 7 (23.1) |
| Anxiety | 321 | (17.4%) | 1,527 (82.6%) | 193 (10.4%) | 81 (4.4%) | 47 (2.5%) | 5 (37.0) |
| Stress and adjustment | 463 | (25.1%) | 1,385 (75.0%) | 235 (12.7%) | 127 (6.9%) | 101 (5.5%) | 4 (22.4) |
| Personality | 350 | (18.9%) | 1,498 (81.1%) | 157 (8.5%) | 98 (5.3%) | 95 (5.1%) | 6 (51.8) |
| Intellectual disability | 105 | (5.7%) | 1,743 (94.3%) | 38 (2.1%) | 40 (2.2%) | 27 (1.5%) | 5 (16.6) |
| Self-harm | 576 | (31.2%) | 1,272 (68.8%) | 254 (13.7%) | 174 (9.4%) | 148 (8.0%) | 3 (48.5) |
| Substance abuse (other) | 415 | (22.5%) | 1,433 (77.5%) | 182 (9.9%) | 160 (8.7%) | 73 (4.0%) | 2 (11.6) |
| Mental Health (Other^1^) | 233 | (12.6%) | 1,615 (87.4%) | 169 (9.1%) | 53 (2.8%) | 11 (0.6%) | 4 (9.1) |

^CMH: Victorian Clinical Mental Health Data^

^ED: Victorian Emergency Department data^

^1 Behavioural syndromes associated with physiological disturbances and physical factors^
