# Supplementary material for: Patterns of homelessness and housing instability and the relationship with mental health disorders among young people transitioning from out-of-home care: Retrospective cohort study using linked administrative data
Source: PLoS One. 2022 Sep 2;17(9):e0274196. doi: 10.1371/journal.pone.0274196 (PMC9439254; doi:10.1371/journal.pone.0274196)
Supplement: S4 Table — (DOCX) [file pone.0274196.s004.docx]

**S4 Table. Mental Health Disorders and ICD-10 Codes**

|  | Mental Health Disorder | Sub-category | ICD 10-Codes | N^1^ (% of Total) | Sub-category |
| --- | --- | --- | --- | --- | --- |
| 1 | Schizophrenia and psychoses | Substance use-psychosis related | F105 F106 F107 F115 F116 F117 F125 F126 F127 F135 F136 F137 F145 F146 F147 F155 F156 F157 F165 F166 F167 F175 F176 F177 F185 F186 F187 F195 F196 F197 | 226 (12%) | 61 (3%) |
|  |  | Schizophrenia | F20* F21* F22* F23* F24* F25* F26* F27* F28* F29* |  | 183 (10%) |
|  |  | Manic/ Bipolar | F30* F31* |  | 76 (4%) |
| 2 | Psychological development or behavioural and emotional disorders with onset usually occurring in childhood and adolescence | Psychological development | F80* F81* F82* F83* F84* F85* F86* F87* F88* F89* | 567 (31%) | 110 (6%) |
|  |  | Child-hood behavioural and emotional | F90* F91* F92* F93* F94* F95* F96* F97* F98* |  | 539 (29%) |
| 3 | Anxiety and Stress-related disorders | PTSD or stress -related (Reactions to severe stress, and adjustment disorders) | F43* | 608 (33%) | 463 (25%) |
|  |  | Anxiety (including anxiety disorders, panic disorder, obsessive compulsive disorder | F40* F41* F42* |  | 321 (17%) |
| 4 | Mood or Depressive Disorders | Depressive episode, recurrent depressive disorder, persistent mood (affective disorder), other mood (affective) disorder, unspecified mood (affective disorder) | F32* F33* F34* F38* F39* | 465 (25%) |  |
| 5 | Personality Disorders |  | F60* F61* F62* F68* F69* | 350 (19%) |  |
| 6 | Intellectual Disability |  | F70* F71* F72* F73* F74* F75* F76* F77* F78* F79* | 105 (6%) |  |
| 7 | Self-harm |  | X6* X7* X80* X81* X82* X83* X84* Z915 R4581 | 576 (31%) |  |
| 8 | Substance use Disorder |  | (F10* F11* F12* F13* F14* F15* F16* F17* F18* F19*) less substance use psychosis | 586 (32%) |  |
| 9 | Substance Use_Other |  | Z721 Z722 X41* X42* X45* Y11* Y12* Y15* T40* T41* T42* T43* T509 T51* | 415 (23%) |  |
| 10 | Organic |  | F00* F01* F02* F03* F04* F05* F06* F07* F08* F09* | 33 (2%) |  |
| 11 | Eating Disorders |  | F50* | 32 (2%) |  |
| 12 | Other Mental Health |  | F44* F45* F48* F51* F52* F53* F54* F55* F56* F57* F58* F59* F63* F64* F65* F66* F67* F99* | 198 (11%) |  |
| 13 | Any Mental Health or substance abuse |  | F0* F1* F2* F3* F4* F5* F6* F7* F8* F9* X6* X7* X80* X81* X82* X83* X84* Z915 R4581 | 1,132 (61%) |  |

^1 Any Diagnosis or Admission from (Hospital, Emergency Department, Clinical Mental Health)^
